# Circulating levels of proinflammatory mediators as potential biomarkers of post-traumatic knee osteoarthritis development

**DOI:** 10.1007/s10195-017-0473-8

**Published:** 2017-10-20

**Authors:** Svetlana B. Panina, Igor V. Krolevets, Natalia P. Milyutina, Alexander B. Sagakyants, Igor V. Kornienko, Anzhelika A. Ananyan, Mikhail A. Zabrodin, Andrey A. Plotnikov, Valeriy V. Vnukov

**Affiliations:** 1 Academy of Biology and Biotechnology, Southern Federal University, 194/1 Stachki Street, 344090 Rostov-on-Don, Russian Federation; 2 Department of Traumatology and Orthopaedics, Rehabilitation and Sport Medicine, Rostov State Medical University, 29 Nakhichevanskii St, Rostov-on-Don, 344022 Russian Federation

**Keywords:** Proinflammatory mediators, Interleukin, Synovial fluid, Post-traumatic knee osteoarthritis, Radiographic severity

## Abstract

**Background:**

The identification of biomarkers of post-traumatic osteoarthritis (PTOA) progression is of clinical importance. The aims of this study were: (1) to assess the abilities of various soluble proinflammatory mediators in plasma to distinguish patients with knee PTOA from controls; (2) to determine the correlations between the mediators in plasma and those mediators in synovial fluid (SF); and (3) to explore the associations of the mediators with radiographic PTOA severity.

**Materials and methods:**

The concentrations of IL-1β, IL-6, IL-18, TNFα, and leptin were measured using ELISA. Nitric oxide was determined as nitrite/nitrate (NO_*x*_) using the Griess reaction.

**Results:**

We included 171 subjects (134 PTOA patients and 37 controls) and excluded patients with rheumatoid arthritis or gout. The ROC curve of plasma NO_*x*_ had the highest AUC, a specificity of 100%, and a sensitivity of 84.4%. The combination of IL-6 and leptin proved to be the most discriminatory, with an AUC value of 0.933, a specificity of 96.7%, and a sensitivity of 85.7%. The levels of NO_*x*_, IL-6, IL-18, and leptin in plasma were significantly correlated with their levels in SF. Leptin levels in both plasma (*p* = 0.036) and SF (*p* = 0.041) and the synovial IL-18 level (*p* = 0.045) were correlated with the Kellgren–Lawrence (KL) grade. Early-stage PTOA (KL 1–2) was associated with a high concentration of IL-1β in plasma before and after (OR 6.235, 95% CI 1.362 to 28.552, *p* = 0.018) adjusting for age, gender, and BMI.

**Conclusions:**

Circulating NO_*x*_ level and a combination of IL-6 and leptin permitted the strongest discrimination of patients with PTOA from controls. PTOA severity was correlated with leptin levels in plasma and SF and with the synovial IL-18 level. Early PTOA was associated with the circulating level of IL-1β.

**Level of evidence:**

III (case–control study).

## Introduction

Osteoarthritis (OA) is a degenerative joint disease that commonly affects weight-bearing joints and is characterized by articular cartilage degradation. Key events occurring during OA pathogenesis include an imbalance of anabolic and catabolic signals driven by cytokine cascades and the production of inflammatory mediators [1–3]. Post-traumatic osteoarthritis (PTOA) subsequent to joint injury accounts for > 12% of the overall disease burden of OA, and is higher in the ankle and knee joints that are most at risk [4]. The identification of biomarkers of PTOA progression is important as therapies are available. Several cytokines have been investigated as possible candidates for biochemical markers [3, 4]. Recent studies have demonstrated correlations of the radiographic grading of OA with many biochemical markers in the blood, including circulating levels of IL-6 and TNF-α [5]. Macrophage-derived cytokines, including IL-1β and TNF-α, are the major players in the cartilage breakdown associated with OA [6]. Both IL-1β and the mechanical loading of cartilage increase NO production by upregulating nitric oxide synthase 2, which may stimulate chondrocyte apoptosis [7, 8]. Higher levels of leptin have been shown to be an independent predictor of radiographic knee changes associated with OA, albeit mainly in women [9]. Uric acid is now considered an active agent in systemic inflammatory and innate immune responses, particularly NLRP3 inflammasome activation, which drives IL-1β and IL-18 production [10].

The aim of the study reported here was to compare the levels of soluble proinflammatory mediators in plasma from patients with early-stage (Kellgren–Lawrence grade 1–2) and late-stage (grade 3–4) PTOA to those in healthy donors, and to assess the abilities of the examined biomarkers to distinguish PTOA patients from non-PTOA controls. In addition, we aimed to determine the correlations between the biomarkers in plasma and those biomarkers in synovial fluid, and the associations of levels of mediators in plasma and SF with radiographic OA severity. Biochemical and immunological biomarkers are often easier to measure than clinical endpoints, so they can be used as ‘surrogate’ endpoints [11]. In our study, we considered occurrence of PTOA after injury to be the primary clinical endpoint and radiographic PTOA severity as the secondary endpoint.

We hypothesized that some of the mediators that contribute to the inflammation process in the joint may serve as informative biomarkers to discriminate between patients with post-traumatic osteoarthritis and healthy subjects, especially patients with early-grade OA. We tried to find statistically significant biomarkers (surrogate endpoints) to correlate with (1) PTOA development (the primary outcome measure) and (2) radiographic OA severity (the secondary outcome measure).

## Materials and methods

### Inclusion and exclusion criteria

We included patients diagnosed with post-traumatic knee osteoarthritis using clinical and radiographic findings. All of the included patients had one affected knee (left or right) and suffered their knee injury ≤ 1 year ago. Nearly all of the patients had endured a meniscus injury that led to PTOA development. Radiographic assessment of severity was performed using the Kellgren–Lawrence grading system [14]. Early knee radiographic OA was considered to be present when the knee had PTOA defined as K/L grade ≤ 2, and late knee radiographic OA was considered to be present when the PTOA was defined as K/L grade ≥ 3.

Exclusion criteria were rheumatoid arthritis, septic arthritis, gout, diabetes, and mechanical axis deviation of the knees. Healthy volunteers who had no clinical or radiological evidence of OA were included in the control group.

### Laboratory methods

Venous blood samples were collected from patients with PTOA and healthy controls after overnight fasting, and were centrifuged and stored immediately at − 80 °C. Synovial fluid was collected in heparin-coated tubes at the time of knee arthrocentesis under sterile conditions by experienced orthopedic physicians. Each SF sample was centrifuged at 3000 rpm to remove any cells and the supernatant was stored at − 80 °C for further biochemical analyses. Freeze–thaw cycles were avoided. The levels of cytokines and leptin were measured using commercially available enzyme-linked immunosorbent assay (ELISA) kits for IL-1β, IL-6, IL-18, TNFα (Vector-Best, Russia), and for leptin (Leptin-DRG, USA) according to the manufacturers’ instructions.

Nitric oxide (NO) was indirectly determined as NO_*x*_ using the Griess reaction described elsewhere [12]. The uric acid level was determined using a commercially available kit (Vital, Russia) and a spectrophotometer (Beckman Coulter DU 800, USA).

### Statistical analysis

Statistical analysis was carried out using the Statistical Package for Social Sciences (SPSS) software, version 23.0. The data are presented as the mean ± SD, median (interquartile range), or frequency. Demographic data for patients and controls were compared by the chi-square test and the Mann–Whitney *U* test, where appropriate.

The levels of mediators were compared using the Kruskal–Wallis test with the post hoc pairwise Dunn’s test and the Mann–Whitney *U* test with Bonferroni correction (*p* value cutoff = 0.05/7). *p* values of less than 0.05 were regarded as statistically significant for the other analyses. One possible limitation of this study is that we did not perform a power analysis beforehand. Instead, we calculated Hodges–Lehmann confidence intervals for the median differences between the groups. This can be especially useful for interpreting negative results [13].

Correlations were calculated using Spearman’s correlation coefficient (*r*). Multinomial logistic regression was used to assess the associations of various factors (mediator levels adjusted for age, gender, and BMI) with the radiographic severity of PTOA.

Receiver operating characteristic (ROC) curves were plotted to compare the discriminatory strengths of different mediators in plasma between PTOA and non-PTOA controls and between early-grade and late-grade PTOA. The sensitivity, specificity, and area under the curve (AUC) were calculated. The best cutoff values were identified using the maximal value of Youden’s index (sensitivity + specificity − 1) as the criterion. We used logistic regression to plot combined ROC curves for combinations of several biomarkers.

## Results

### Subjects

The baseline characteristics of the patients with PTOA (early-grade and late-grade groups) and healthy controls are shown in Table 1. There were no statistically significant differences in age, BMI, or gender between the PTOA patients and the controls. We excluded 15 patients from the study due to the reasons listed above.

**Table 1:** Demographic characteristics of patients with PTOA and healthy individuals

Characteristics	Healthy donors (*n* = 37)	PTOA (*n* = 134)	p*	KL 1–2 (*n* = 103)	KL 3–4 (*n* = 31)
Age (years)	40.19 ± 10.11	43.63 ± 15.14	0.26	40.88 ± 14.92	52.77 ± 12.15
Gender (male/female)	15/22	60/74	0.65	50/53	10/21
BMI (kg/m^2^)	26.07 ± 2.63	28.15 ± 5.59	0.15	28.18 ± 6.0	28.05 ± 4.16

Demographic data are shown as M±SD

* *p* value for the comparison of healthy subjects with PTOA patients

### Levels of proinflammatory mediators in plasma and SF

**Fig. 1:**
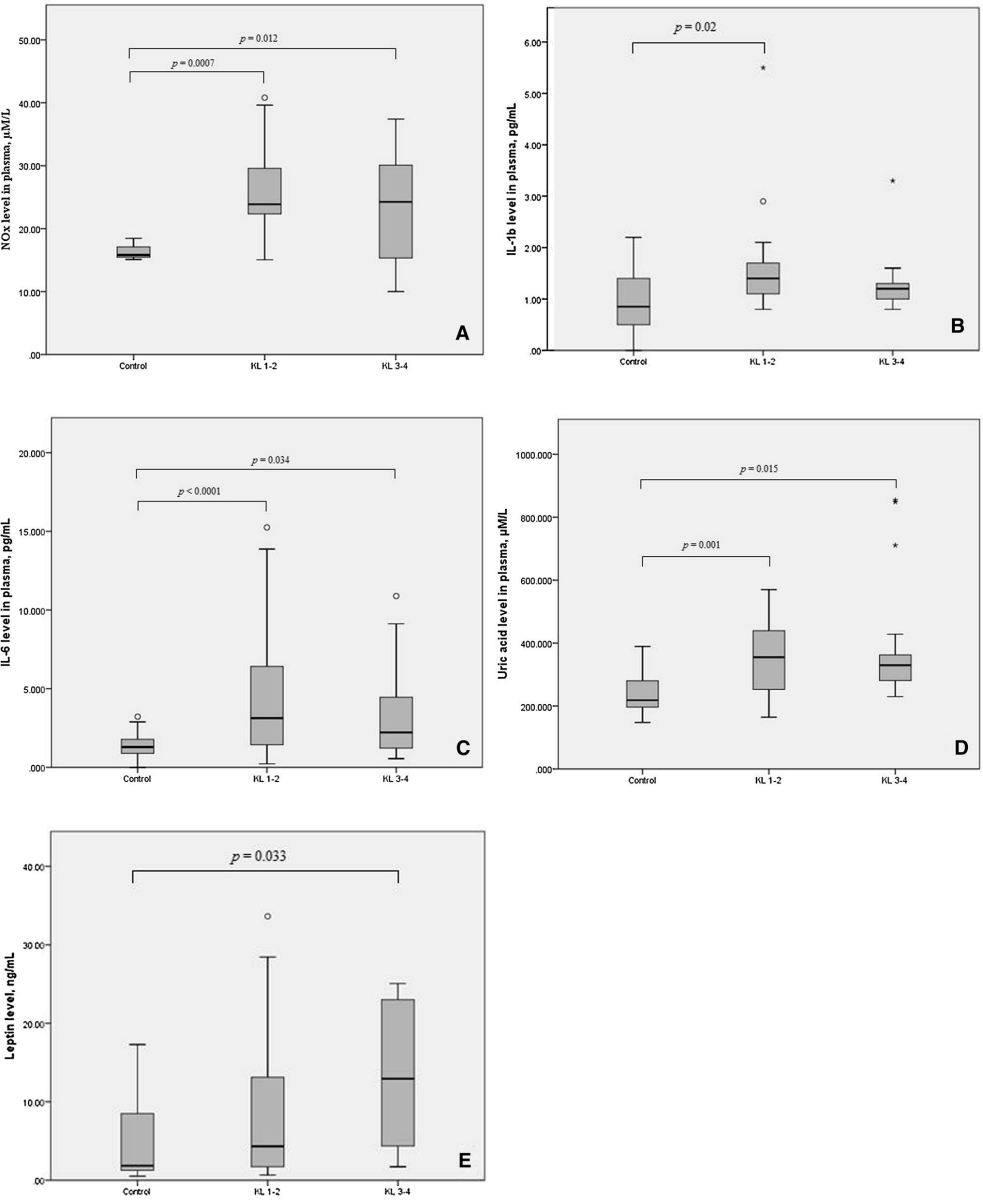
Elevated plasma levels of soluble proinflammatory mediators in PTOA patients (early-stage KL 1–2; late-stage KL 3–4) compared with controls (Kruskal–Wallis test with the post hoc pairwise Dunn’s test). **a** NO_x_ level; **b** IL-1β level; **c** IL-6 level; **d** uric acid level; **e** leptin level.* Boxes* indicate the 25 and 75% percentiles;* whiskers* indicate the minimum to maximum values;* bars* indicate the median;* circles* and* asterisks* indicate outliers

As shown in Table 2 and Fig. 1, the levels of nitrite/nitrate, IL-1β, IL-6, uric acid, and leptin significantly differed between patients with early-stage PTOA, patients with late-stage PTOA, and controls. At the same time, the levels of these mediators in the synovial fluid did not differ between patients with early and late disease. Furthermore, analyses of Hodges–Lehmann estimators for the median differences between the groups (Table 3) yielded nearly the same pattern of significant differences as the Mann–Whitney *U* test or Dunn’s post hoc test (Table 2) did. The deviations from this pattern were the level of nitrite/nitrate in plasma of patients with late PTOA and the TNFα level in plasma of patients with early PTOA when compared with the controls. Therefore, these results should be confirmed using larger sample size.

**Table 2:** Plasma and synovial fluid levels of soluble mediators in osteoarthritis patients and controls

Mediator	Healthy controls	Total PTOA	KL 1–2	KL 3–4	*p*
Plasma					
NO_*x*_ (μM/L)	15.81 (15.47 to 17.11)	23.88 (22.17 to 29.63)	23.88 (22.35 to 29.58)^a^	24.26 (15.33 to 30.1)^a^	**< 0.0001**
IL-1β (pg/mL)	0.85 (0.5 to 1.4)	1.25 (1.0 to 1.7)	1.4 (1.1 to 1.7)^a^	1.2 (1.0 to 1.3)	**0.0185**
IL-6 (pg/mL)	1.29 (0.89 to 1.78)	2.75 (1.22 to 6.0)	3.13 (1.44 to 6.42)^a^	2.22 (1.22 to 4.67)^a^	**0.0001**
IL-18 (pg/mL)	147.0 (113.0 to 209.0)	138.17 (88.0 to 186.0)	147.17 (88.0 to 196.0)	113.63 (83.5 to 140.17)	0.50
TNFα (pg/mL)	2.29 (1.0 to 3.43)	0.71 (0 to 2.43)	0.43 (0 to 1.43)	2.21 (0.36 to 5.93)	0.111
Uric acid (μM/L)	218.51 (196.5 to 280.03)	344.99 (265.93 to 428.47)	355.18 (252.6 to 439.39)^a^	329.69 (280.87 to 362.35)^a^	**0.0017**
Leptin (ng/mL)	1.85 (1.26 to 8.50)	5.81 (1.76 to 19.19)	4.32 (1.71 to 13.13)	12.94 (4.36 to 23.0)^a^	**0.0346**
Synovial fluid					
NO_*x*_ (μM/L)	–	24.23 (22.38 to 28.41)	23.99 (22.34 to 27.29)	26.27 (23.13 to 29.05)	0.26
IL-1β (pg/mL)	–	1.45 (1.2 to 2.0)	1.5 (1.1 to 3.8)	1.3 (1.2 to 1.7)	0.60
IL-6 (pg/mL)	–	3054 (346 to 6000)	3256 (392 to 6000)	1597.5 (300 to 6000)	0.50
IL-18 (pg/mL)	–	68.13 (54.5 to 80.0)	67.13 (53.0 to 81.0)	75.38 (69.13 to 77.63)	0.42
TNFα (pg/mL)	–	1.43 (0.14 to 3.14)	1.43 (0.43 to 5.14)	1.0 (0.07 to 2.29)	0.34
Uric acid (μM/L)	–	288.24 (218.12 to 380.0)	289.91 (211.12 to 394.02)	283.02 (234.28 to 361.75)	0.99
Leptin (ng/mL)	–	4.98 (3.05 to 18.75)	4.91 (2.77 to 18.13)	9.67 (5.0 to 22.13)	0.18

Data are presented as median (25 to 75% quartiles). The plasma levels of mediators were compared by performing a Kruskal–Wallis test of three groups—the healthy, KL 1–2, and KL 3–4 subgroups—and a subsequent Dunn’s test. The SF levels of mediators were compared by performing a Mann–Whitney *U* test between the KL 1–2 and KL 3–4 patient subgroups

Bold* p* values are statistically significant (*p* < 0.05)

^a^Significantly different (*p* < 0.05) according to the post hoc Dunn’s test when the particular group was compared with the control group

**Table 3:** Hodges–Lehmann estimates for the confidence intervals for the median differences between the groups

Mediator	Control vs. KL 1–2	Control vs. KL 3–4	KL 1–2 vs. KL 3–4
Plasma			
NO_*x*_	− 8.02 (− 12.05 to − 6.55)*	−** 7.48 (**− **13.50 to 0.19)**	2.20 (− 2.68 to 6.97)
IL-1β	− 0.6 (− 1.0 to − 0.2)*	− 0.4 (− 0.8 to 0.1)	0.2 (− 0.2 to 0.7)
IL-6	− 1.97 (− 3.88 to − 0.99)*	− 1.0 (− 2.19 to − 0.22)*	0.82 (− 0.53 to 2.47)
IL-18	13.88 (− 30.0 to 54.0)	45.71 (− 13.0 to 107.75)	26.71 (− 31.0 to 101.75)
TNFα	1.0 (0.0 to 2.286)*	− 0.358 (− 3.857 to 1.572)	− 0.857 (− 4.286 to 0.429)
Uric acid	− 100.16 (− 170.29 to − 44.15)*	− 95.13 (− 156.99 to − 49.66)*	1.59 (− 65.57 to 70.96)
Leptin	− 2.18 (− 8.65 to 0.05)	− 7.71 (− 19.64 to − 1.0)*	− 3.28 (− 12.31 to 2.0)
Synovial fluid			
NO_*x*_	–	–	− 0.95 (− 3.26 to 0.84)
IL-1β	–	–	0.1 (− 0.3 to 2.1)
IL-6	–	–	0 (− 228.56 to 2404.0)
IL-18	–	–	− 6.5 (− 21.5 to 16.25)
TNFα	–	–	0.429 (− 1.143 to 3.143)
Uric acid	–	–	− 0.02 (− 58.56 to 63.07)
Leptin	–	–	− 1.91 (− 7.29 to 7.19)

Data are presented as Hodges–Lehmann estimator (95% CI lower bound to upper bound). Cases where the null hypothesis was rejected are labeled with asterisks (*). Cases which deviate from the pattern of significant differences defined by the Mann–Whitney *U* test or Dunn’s test are highlighted in bold

### Correlation between the levels in plasma and SF

The levels of NO_*x*_, IL-6, IL-18, and leptin in plasma were significantly correlated with those in synovial fluid: *r* = 0.585, *p* < 0.00001; *r* = 0.457, *p* = 0.0015; *r* = 0.299, *p* = 0.044; *r* = 0.864, *p* < 0.00001, respectively. As expected, the BMI of patients with PTOA was found to be strongly correlated with the leptin levels in plasma (*r* = 0.723, *p* < 0.000001) and SF (*r* = 0.604, *p* = 0.0003).

### Potential biomarkers to distinguish PTOA patients from non-PTOA controls

**Fig. 2:**
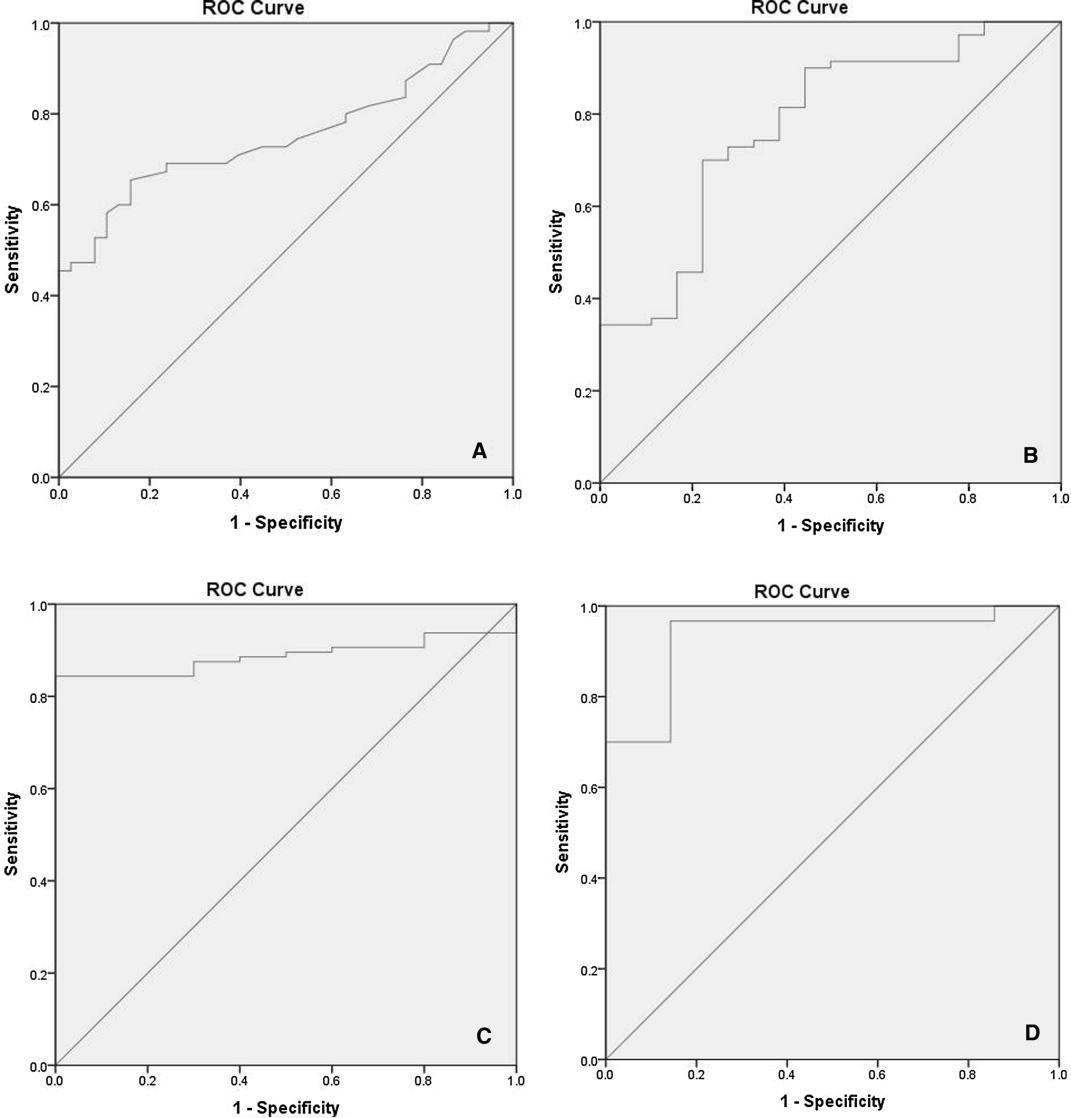
The most statistically significant ROC curves of PTOA markers in plasma. The* diagonal line* is the reference line. **a** IL-6 level; **b** uric acid level; **c** NO_*x*_ level; **d** levels of IL-6 and leptin

All of the measured mediators in plasma except for IL-18 and TNFα gave statistically significant results in ROC curve analysis (Table 4). Figure 2a–c show the three most significant ROC curves of individual markers in plasma (NO_*x*_, IL-6, uric acid).

**Table 4:** ROC curve characteristics: circulating proinflammatory mediators as potential biomarkers of PTOA progression

Parameter	AUC (95% CI)	*p*	Cutoff	Sensitivity (%)	Specificity (%)
Plasma (PTOA vs control)					
NO_*x*_	0.888 (0.826–0.949)	< 0.00001	> 18.73 μM/L	84.4	100
Uric acid	0.774 (0.653–0.894)	< 0.00001	> 280.45 μM/L	70	77.8
IL-6	0.753 (0.655–0.851)	< 0.00001	> 2.06 pg/mL	65.5	84.2
IL-1β	0.730 (0.587–0.872)	0.007	> 1.15 pg/mL	68.2	69.2
Leptin	0.694 (0.535–0.852)	0.028	> 3.59 ng/mL	65.7	75

Statistically significant ROC curves are shown

We tried to calculate whether a combination of two markers that both showed correlations between their plasma and SF levels could enhance the predictive power of the test compared to the use of individual markers. By applying logistic regression to the combination of IL-6 and leptin, it was possible to show that this combination was the most discriminatory, with an AUC value of 0.933, 95% CI 0.839–1.0, *p* < 0.00001, a specificity of 96.7%, and a sensitivity of 85.7% (Fig. 2d). However, this combination could not discriminate patients with early-stage PTOA from those with late-stage disease (*p* > 0.05).

### Associations of the levels of proinflammatory mediators with radiographic PTOA progression

Correlation analysis demonstrated that PTOA grade was significantly associated with leptin level in both plasma (*r* = 0.355, *p* = 0.036) and synovial fluid (*r* = 0.362, *p* = 0.041) as well as with synovial IL-18 level (*r* = 0.297, *p* = 0.045). Further comparative analysis between the KL 1–2 and KL 3–4 PTOA subgroups revealed that BMI is not responsible for the correlation of leptin with KL grade because BMI did not differ between these subgroups (*p* = 0.722).

Multinomial logistic regression analysis with KL grade (0 vs. 1–2 vs. 3–4) as the dependent variable and the quartiles of the mediators as independent variables indicated that low levels of leptin (first quartile, 0.524–1.652 ng/mL) in plasma significantly reduced the probability of early-stage PTOA development (OR 0.083, 95% CI 0.008–0.849, *p* = 0.036) and the probability of late-stage PTOA development (*p* < 0.00001) with respect to the control group (KL 0). Levels of plasma IL-6 and uric acid were positively associated with an early and a late KL grade (*p* < 0.00001). However, these associations disappeared after adjusting for gender, age, and BMI. Multinomial logistic regression analysis showed that early PTOA was associated with a high IL-1β plasma concentration (OR 4.5, 95% CI 1.189–17.03, *p* = 0.027). After adjusting for age, gender, and BMI, this association was found to be stronger: OR 6.235, 95% CI 1.362–28.552, *p* = 0.018. None of the associations between quartiles of mediators in SF and progression of radiographic knee OA (KL 1–2 vs. KL 3–4) were significant before or after adjusting for age, gender, and BMI.

## Discussion

Investigations of potential biomarkers for OA diagnosis are still at the forefront of research into OA [3]; however, biomarkers of PTOA progression have not been as extensively studied. The principal findings of this study are that some circulating biomarkers, namely nitrite/nitrate level, the combination of IL-6 and leptin, as well as IL-1β, may be used to predict the occurrence of PTOA (early PTOA in the case of IL-1β). Leptin levels in plasma and SF and the IL-18 level in SF are associated with PTOA progression.

Some studies have speculated that to prevent the development of PTOA in injured knees, it could be important to neutralize proinflammatory cytokines such as IL-6, IL-8, and IL-1 [15, 16]. Increased levels of IL-6 and IL-8 have been observed following anterior cruciate ligament injury [15–18]. The present results indicate that the plasma level of IL-6 was significantly higher in patients with early- and late-stage PTOA than in healthy controls, and that the level of IL-6 in plasma was correlated with the synovial IL-6 level.

Circulating IL-1β was found to be associated with early-stage PTOA even after adjustment for age, gender, and BMI (Fig. 1). This is consistent with the results of a study [19] in which IL-1β did not appear to play a prominent role in end-stage OA, and the findings of a study [20] in which higher synovial IL-1β expression was observed in patients with less severe OA (KL score 2 or 3 vs. 4). The concentrations of IL-1β and IL-6 were higher than basal levels in the synovial fluid of patients with acute injury of the knee joint [17].

We did not find a significant difference in TNFα level between PTOA patients and controls, or between patients with early and late disease. In the literature, TNFα was not detectable or was only detected at low levels in synovial fluid from patients with a post-traumatic wrist as well as primary knee osteoarthritis [19]. The SF environment in an anterior cruciate ligament injury has been shown to be mainly anabolic and inflammatory, with increased levels of IL-1 and IL-6 and significantly decreased levels of TNFα [21].

ROC curve analysis indicated that a combination of plasma IL-6 and leptin yielded the best AUC parameters for discriminating the PTOA group from controls. We found a positive correlation between the leptin levels in SF and those in plasma, and correlations between these parameters and the radiographic severity of OA, consistent with the study of Ku et al. [22]. These findings have particular significance because the review of Kluzek et al. [9] did not identify any studies that looked at the role of leptin specifically in the development of post-traumatic knee osteoarthritis. Furthermore, leptin may be considered a marker of late-stage disease (Fig. 1). Interestingly, similar to IL-6, leptin released by adipose tissue has been shown to induce vascular permeability and to stimulate angiogenesis via the growth factors FGF-2 and VEGF [23]. It is known that angiogenesis and inflammation are closely integrated processes in OA, which may affect disease progression and pain [24].

Leptin has previously been shown to induce NO production through the activation of the eNOS phosphorylation pathway [25] and by enhancing the inductive action of IL-1 on iNOS [26]. NO is rapidly oxidized in vivo into nitrates and nitrites and then excreted; however, we did not find an association between urinary nitrate levels and knee OA [27]. Consistent with the study of Ersoy et al. [28], our results demonstrated NO_*x*_ elevation in PTOA, especially in individuals with early PTOA (Fig. 1). The ROC curve of the NO_*x*_ level in plasma had high discriminatory power. In essence, NO_*x*_ may be considered a marker of early-stage PTOA.

Synovial IL-18 was significantly correlated with PTOA severity; plasma uric acid levels were elevated in PTOA patients. Denoble et al. demonstrated that synovial fluid uric acid was correlated with the synovial levels of cytokines IL-1β and IL-18, which are known to be produced by uric-acid-activated inflammasomes, and they showed an association of synovial fluid IL-18 with OA progression [29]; the latter association is consistent with our results. An association of IL-18 with pain in OA has been reported [29]. Serum uric acid levels were associated with KL grade and pain in the subjects, and in females with knee OA [30]. Intriguingly, leptin was shown to enhance IL-18 secretion from human monocytes by modulating caspase-1 inflammasome function [31]. However, we did not observe a correlation of IL-18 with the leptin levels in plasma or SF (*p* > 0.05).

There are several potential limitations of the present study. Most importantly, we did not measure levels of proinflammatory mediators in SF of healthy donors due to ethical concerns, so we could not compare the levels in SF of PTOA patients with those in controls. Second, we enrolled a relatively small number of patients with late-grade PTOA. Actually, this difference between the number of patients in the early PTOA group and the number in the late PTOA group has a statistical basis: a population-based study of OA in North Carolina showed that 28% of the participants had radiographic knee OA (K/L radiographic grade ≥ 2) but only 8% of them had severe radiographic knee OA (grade 3–4) [32]. However, the results obtained should be confirmed using a larger sample.

In summary, patients with early- and late-stage post-traumatic knee osteoarthritis have increased levels of circulating proinflammatory nitrite/nitrate (NO_*x*_), IL-6, and uric acid compared with healthy subjects. Circulating levels of NO_*x*_, IL-1β, IL-6, uric acid, leptin, and the combination of IL-6 and leptin may be potential diagnostic tests according to the results of ROC curve analyses. The levels of NO_*x*_, IL-6, IL-18, and leptin in plasma were significantly correlated with those in SF. PTOA severity was correlated with leptin levels in both plasma and SF and with the synovial IL-18 level. Multinomial logistic regression showed that a higher level of IL-1β in plasma was associated with early-stage PTOA after adjusting for age, gender, and BMI. Further studies on proinflammatory mediators are necessary to facilitate the development of new diagnostic approaches to prevent post-traumatic osteoarthritis.
